# Application of Short Pre-enrichment, and Double Chemistry Real-Time PCR, Combining Fluorescent Probes and an Intercalating Dye, for Same-Day Detection and Confirmation of *Salmonella* spp. and *Escherichia coli* O157 in Ground Beef and Chicken Samples

**DOI:** 10.3389/fmicb.2020.591041

**Published:** 2020-10-09

**Authors:** Alejandro Garrido-Maestu, Sarah Azinheiro, Foteini Roumani, Joana Carvalho, Marta Prado

**Affiliations:** ^1^Food Quality and Safety Research Group, International Iberian Nanotechnology Laboratory, Braga, Portugal; ^2^Department of Analytical Chemistry, Nutrition and Food Science, School of Veterinary Sciences, University of Santiago de Compostela, Santiago de Compostela, Spain

**Keywords:** melt curve analysis, *Salmonella* spp., *Escherichia coli* O157, intercalating dye, same-day detection, hydrolysis probe

## Abstract

Molecular methods, particularly those based on real-time PCR (qPCR), have become a popular approach to detect pathogens in food samples. This technique may take advantage of hydrolysis fluorescent probes for increased specificity. Even though suitable, this approach loses the capacity of performing result confirmation by melt curve analysis. In the current study, we developed an alternative approach, combining fluorescent probes along with an intercalating dye (SYBR Green) in order to simultaneously detect, and confirm the result, of two foodborne pathogens (*Salmonella* spp. and *Escherichia coli* O157). This new approach named double chemistry qPCR was combined with a short pre-enrichment in order to obtain a multiplex “same-day” detection method for the selected pathogens. The evaluation of the novel method in spiked food samples (ground beef and chicken breast) obtained values of relative sensitivity, specificity, and accuracy higher than 95%, and Cohen’s kappa of 0.92, with a Limit of Detection_95_ below 5 cfu/25 g, demonstrating its reliability. In addition to this, the method was challenged by inoculating heat-stressed bacteria as well as dead ones. It was observed that it was also possible to detect stressed bacteria with an initial inoculation level below 10 cfu/25 g. Also, it was noticed that high initial concentration of either pathogen (higher than 10^4^ cfu/25 g) was needed in order to generate false positive results due to the presence of dead bacteria, thus the method presents potential for its application in the specific detection of live microorganisms.

## Introduction

Molecular methods, particularly those based on DNA amplification, have become a very popular approach when fast and accurate results are needed in the food industry. Real-time PCR (qPCR) is one of the most widely accepted techniques, due to its high sensitivity, specificity, and capacity to detect the amplified fragments in real time without the need of additional manipulation (Kokkinos et al., 2014; Chapela et al., 2015). Also, it can take advantage of fluorescent probes for improved specificity. Out of these, the most commonly implemented are hydrolysis probes, due to their relatively simple design and reduced cost. A limitation of these probes is that melt curve analysis cannot be performed as the probe is degraded in the process of amplification. This issue may be overcome by selecting a hybridization probe instead, like the molecular beacons (MBs) or “adjacent probes” (Marras et al., 2006), but their design, particularly in the case of the MB, tends to be more complex as not only the specific target sequence has to be considered, but also the stem and the secondary structure formed (Zheng et al., 2015), and the cost is higher. Additionally, in the melting values obtained only correspond to that of the probes, and not to the complete amplified fragment (O’Grady et al., 2008; Chakravorty et al., 2010).

Nowadays, foodborne pathogens continue to be a major threat for human health. Among them, two of the most commonly reported bacterial pathogens are *Salmonella* spp. and Shiga Toxin-Producing *Escherichia coli* (STEC). According to the European Food Safety Authority, in 2017 a total of 91,662 confirmed salmonellosis cases were reported in Europe, as well as 6,073 STEC infections, being serogroup O157 the most commonly reported (31.9%). Overall the hospitalization rate was around 40%, what further highlights the health and economic impact of these pathogens (EFSA and ECDC, 2018).

Current reference methods for the detection of these bacteria in food samples are culture-based. Among these, the most extended ones are those described by the ISO and the FDA (ISO, 2002, 2003; Andrews et al., 2011; Feng et al., 2011). It is worth to mention that the FDA also provides a molecular-based method for the detection of *E. coli* O157:H7. All these methods have demonstrated to be highly reliable, and in the particular case of *E. coli*, novel approaches were included in the method such as immunomagnetic separation to concentrate specific serotypes, or implementation of qPCR for accurate detection, but lacks the capacity of result confirmation by melt curve as commented above. It may also be noted that all the mentioned reference methods begin with a sample enrichment/pre-enrichment what generates an overall delay of the results regardless the implementation of qPCR or not. A high number rapid methods have been reported, many based on PCR/qPCR, and even included in multi-center validation trials, but they still rely on sample enrichment to increase the concentration of the target bacteria to detectable numbers, what in the best case scenario makes them next-day detection approaches (Abdulmawjood et al., 2003, 2004; Malorny et al., 2003, 2004; Cheng et al., 2009, 2015). In this sense, recent studies have reported that an appropriate sample pre-treatment can significantly reduce the time of analysis by directly tacking the major bottleneck, the enrichment step. In the study published by Fachmann et al. (2017), they indicated that a short pre-enrichment (SpEn; 3 h) could allow for a sensitive detection of *Salmonella* spp. in meat samples. Likewise, Garrido-Maestu et al. (2020) followed a similar approach and also demonstrated that the methodology could be applied, with minor modifications, for the rapid detection of *E. coli* O157 in meat and salad samples. These studies provide real same-day detection of the pathogens, but have only targeted one single bacterial species, thus presenting a lower throughput.

In the current study, the development of a same-day detection methodology based on SpEn for the simultaneous detection of *Salmonella* spp. and *E. coli* O157 was developed. Additionally, the multiplex detection of the bacteria was performed by a novel qPCR approach named double chemistry (DC-qPCR) as it combines hydrolysis probes along with an intercalating dye (SYBR Green) in order to simultaneously detect and confirm the results.

## Materials and Methods

### Bacterial Strains and Culture Media

*Escherichia coli* O157 WDCM 00014 and *Salmonella* Typhimurium WDCM 00031 (World Data Centre for Microorganisms) were selected as the reference strains for spiking experiments. Fresh cultures of both bacteria were prepared inoculating one single colony in 4 mL of Nutrient Broth (NB, Biokar diagnostics S.A., France) and incubated overnight at 37°C. After incubation, 100-fold serial dilutions were performed and plated on Tryptic Soy Agar (TSA, Biokar diagnostics S.A., France). The plates were incubated overnight at 37°C and counted to obtain a reference value of viable bacteria.

The sample SpEn was performed in buffered peptone water (BPW, Biokar diagnostics S.A., France) supplemented with 0.4% (w/v) of Tween 80 (Sigma-Aldrich, St. Louis, United States). The confirmation of the results obtained by the molecular method was performed by a culture-based approach. To this end, for the confirmation of *Salmonella* spp. Rappaport Vassiliadis Soya (RVS, Biokar diagnostics S.A., France), xylose lysine deoxycolate (XLD, Biokar diagnostics S.A., France), and ChromAgar™ *Salmonella* Plus (CHROMagar Microbiology, Paris, France) were used. Regarding *E. coli* O157, the media used were modified Tryptic Soy Broth with novobiocin (mTSBn, Biokar diagnostics S.A., France), Sorbitol MacConkey with Cefixime and Tellurite (CT-SMAC, Sigma-Aldrich, St. Louis, United States), and ChromAgar™ O157 (CHROMagar Microbiology, Paris, France). Details about the procedure followed are provided below in M&M 2.6.

### Short Pre-enrichment

Two different food samples were included in the current study, ground beef and chicken breast. The protocol of analysis followed was adapted from Garrido-Maestu et al. (2020). Briefly, 25 g were mixed with 25 mL of BPW, with 0.4% of Tween 80, pre-warmed at 37°C in a filter bag for stomacher, the matrix was hand-massaged and incubated at 37°C for 3 h with constant agitation (200 rpm). After the 3 h, the enriched sample was recovered from the filter side to remove large food particles, and centrifuged at 8,960 *g* for 10 min, the supernatant was discarded, and the pellet was resuspended in 45 mL of protease buffer, and incubated horizontally at 37°C for 10 min at 200 rpm. After digestion, the samples were centrifuged as described above. The pellet was resuspended in 45 mL of washing buffer. This was followed by a new centrifugation step at 8,960 *g* for 10 min. The new pellet was recovered in 1.5 mL of washing buffer, transferred to a clean tube, and centrifuged at 11,000 *g* for 5 min. Finally, the bacterial pellet was rinsed with 1 mL of phosphate buffered saline (PBS), and centrifuged once more at 11,000 *g* for 5 min. The clean pellet was used for DNA extraction.

### DNA Extraction

The protocol described by Garrido-Maestu et al. (2020) was followed. The bacterial pellet recovered after the SpEn was resuspended in 200 μL of 6% Chelex®100 (w/v; Bio-Rad Laboratories, Inc., United States), supplemented with 25 μL of Proteinase K (Macherey-Nagel, Düren, Germany), incubated at 56°C for 15 min, and this was followed by a thermal lysis at 99°C for 10 min. Both incubation steps were performed with constant agitation at 1,400 rpm in a Thermomixer comfort (Eppendorf AG, Germany). Finally, the samples were centrifuged at 11,000 *g* at 4°C for 5 min, and the supernatants were transferred to new clean tubes and sterile tubes for storage at 4°C until analysis.

### Multiplex DC-qPCR

The detection of both bacterial pathogens was performed by multiplex qPCR. To this end, the detection of *Salmonella* spp. was performed targeting the *ttr* gene with the primers (200 nM) and probe (150 nM) described by Garrido-Maestu et al. (2017), regarding *E. coli* O157, and the *rfbE* gene (Garrido-Maestu et al., 2020) was targeted (500 nM primers and 250 nM probe). In addition to these, a non-competitive internal amplification control (IAC) was also included to avoid false negative results due to reaction inhibition (100 nM primers and probe, and 7 × 10^3^ copies of IAC DNA; Garrido-Maestu et al., 2019). All primers and probes were purchased from Integrated DNA Technologies (Integrated DNA Technologies Inc., Leuven, Belgium), and are provided in Table 1.

**Table 1:** Primers and probes.

	Sequence (5'→3')	Modification	Reference
ttr-P3F	GGC TAA TTT AAC CCG TCG TCA G	-	(Garrido-Maestu et al., 2018b)
ttr-P3R	GTT TCG CCA CAT CAC GGT AGC	-	
ttr-P3P	AAG TCG GTC TCG CCG TCG GTG	NED/MGBNFQ	
O157-rfbE-F	TCA ACA GTC TTG TAC AAG TCC AC	-	(Garrido-Maestu et al., 2020)
O157-rfbE-R	ACT GGC CTT GTT TCG ATG AG	-	
O157-rfbE-P	ACT AGG ACC//GCA GAG GAA AGA GAG GAA	FAM/ZEN/IABkFQ	
NC-IAC-F	AGT TGC ACA CAG TTA GTT CGA G	-	(Garrido-Maestu et al., 2019)
NC-IAC-R	TGG AGT GCT GGA CGA TTT GAA G	-	
IAC-P	AGT GGC GGT//GAC ACT GTT GAC CT	YY/ZEN/IABkFQ	(Garrido-Maestu et al., 2018a)

YY, Yakima Yellow; IABkFQ, Iowa Black®FQ; ZEN (secondary, internal quencher) are trademarks from IDT.

The qPCR was performed in a final reaction volume of 20 μL, composed of 10 μL of TaqMan®Fast Advanced Master Mix supplier (Applied Biosystems™, Foster City, CA, USA), with the primer/probe concentrations specified above, 1 μL of 10X Sybr Geen I dissolved in DMSO (SG, Invitrogen™, Carlsbad, CA, United States), and 3 μL of template DNA, and the remaining volume was filled with sterile milliQ water.

The thermal profile consisted of a uracil-DNA glycosylase (UDG) treatment for 2 min at 50°C. This was followed by 2 min at 95°C for polymerase activation, and then 40 cycles of dissociation at 95°C for 1 s, and annealing-extension at 61°C for 20 s. This was continued by a melt curve stage, where the temperature was increased to 95°C for 1 s, decreased to 70°C for 20 s, and increased back to 95°C at a rate of 0.1°C/s. The analysis was performed in a QuantStudio 5 real-time PCR system with the QuantStudio™ Design and Analysis Software v1.4.3 (Applied Biosystems™, Foster City, CA, United States). Positive samples will be considered those providing amplification of the corresponding fluorophore, and with a melting peak at the expected temperature. In the same way, negative samples will lack amplification of the specific fluorophores and/or will not present the expected melting peak, in addition of having amplification for the NC-IAC with its own specific melting peak.

Correct performance of the novel approach was confirmed evaluating the amplification efficiency and the dynamic range of the multiplex reaction using 10-fold dilutions of pure bacterial DNA mixed in equal concentrations. Three biological replicates with three technical replicates for each concentration were analyzed (total of nine data for each point). The amplification efficiency was calculated with the following formula: *E* = 10^−1/b−1^, where “*E*” is the efficiency, and “b” the slope of the curve obtained (González-Escalona et al., 2009).

### Confirmation Procedure

A total of 100 μL of the SpEn were transferred to 10 mL of RVS and mTSBn, which were incubated at 42 and 37°C overnight respectively. After selective enrichment, a loopful of RVS was streaked on XLD and ChromAgar™ *Salmonella*. The mTSBn was streaked on CT-SMAC and ChromAgar™ O157. All plates were incubated overnight at 37°C, and screened the following day for typical colonies of the corresponding pathogen.

### Evaluation of the Methodology

The evaluation of the proposed method was performed in spiked food samples. The 95% Limit of Detection (LOD_95_) was determined. This was followed by the evaluation of its fit-for-purpose attending to its relative sensitivity, specificity, and accuracy (SE, SP, and AC), as well as its positive and negative predictive values (PPV/NPV) and its Cohen’s kappa (*k*). The determination of all this parameters is detailed below.

#### Ninety-Five Percent Limit of Detection

The LOD_95_ was calculated according to Wilrich and Wilrich (2009). Thirty-two samples were spiked, 16 with each pathogen. The samples were divided in four groups of four samples, and each group was inoculated with decreasing concentrations of the corresponding pathogen, with the goal of reaching a concentration with positive and negative results (starting from an inoculation level between 10 and 10 × 10 cfu/25 g, down to a range between 1 and 10 cfu/25 g). The spiking was performed from fresh cultures prepared as detailed in M&M 2.1, as well as the serial dilutions and plating to determine the reference values of viable bacteria.

#### Fit-for-Purpose

Additional samples were inoculated with different concentrations in order to evaluate the performance of the method. These were classified attending to the obtained and expected result as positive/negative agreements if they match the expected result (PA/NA) or deviations if they did not match (PD/ND). These data obtained were used for the calculation of the relative sensitivity, specificity, and accuracy (SE, SP, and AC), along with the PPV/NPV and the Cohen’s *k*, as described elsewhere (Tomas et al., 2009; Anderson et al., 2011; NordVaL, 2017).

### Extended Study

#### Stressed Bacteria

In order to determine the performance of the methodology in a more realistic scenario, two sets of four samples were inoculated with thermally stressed bacteria. These were generated diluting 1/100 in PBS a fresh culture, prepared as described in M&M 2.1, and then heat-treat the dilution at 60°C for 10 min with constant agitation (1,000 rpm). The stressed bacteria were 100-fold serially diluted and inoculated at a final concentration below 10 cfu/25 g. A set of four-spiked samples were stored refrigerated (4–8°C) for 24 h, and another four were stored for 48 h. On the corresponding day, all samples were analyzed following the methodology described above.

#### Dead Bacteria

A final test was conducted in order to determine the effect of dead target bacteria in the final result. To do so, both target species were inactivated. The protocol followed consisted on taking 1 mL of a fresh culture of the corresponding microorganism, prepared as described in M&M 2.1. This was centrifuged at 16,000 *g* for 5 min. The supernatant was eliminated, and the bacterial pellet was resuspended in 1 mL of isopropanol 70% and heated at 92°C for 15 min. Finally, the dead bacterial suspension was centrifuged again at 16,000 *g* for 5 min, the isopropanol was removed, and the pellet was resuspended in 1 mL of PBS. A loopful was streaked on TSA and incubated at 37°C for 24 h to assure correct inactivation of both pathogens. The dead bacteria were used to inoculate four pairs of samples, eight in total, with increasing concentrations ranging from 10^3^ to 10^6^ cfu/25 g. The results obtained were introduced in the LOD model of Wilrich and Wilrich, in order to determine which was the minimum bacterial concentration needed to generate a false positive result due to the presence of dead microorganisms.

## Results

### Determination of the Amplification Efficiency of the Multiplex DC-qPCR

**Figure 1:**
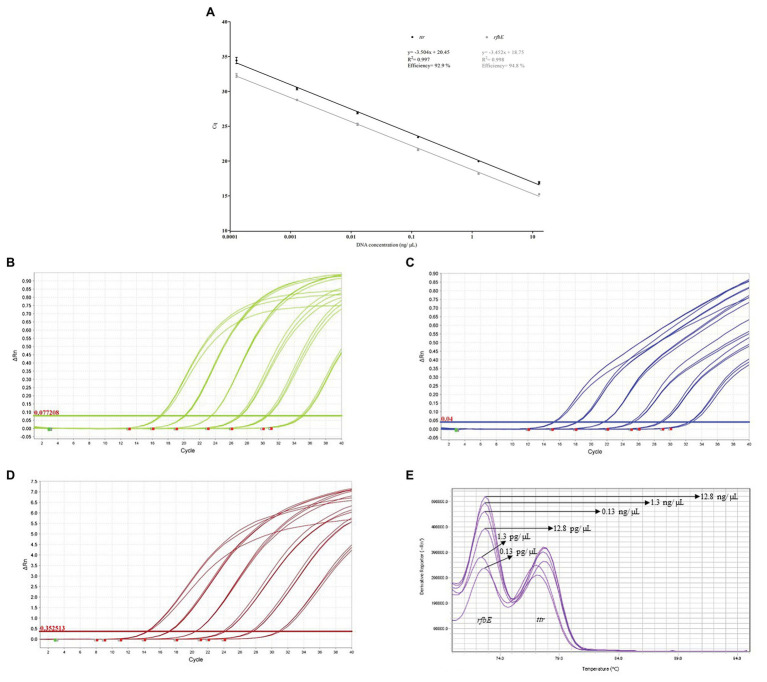
**(A)** Multiplex double chemistry qPCR (DC-qPCR) amplification efficiency. **(B)** *ttr* amplification plots. **(C)** *rfbE* amplification plots. **(D)** SG amplification plots. **(E)** Melt curves generated after multiplex DR-qPCR.

The calculation of the multiplex DC-qPCR efficiency obtained values of 92.9 and 94.8% for *ttr* and *rfbE*, respectively. Additionally, the *R*^2^ was determined to be 0.997 and 0.998. These results are graphically depicted in Figure 1A. In Figures 1B–D, the amplification plots obtained for *ttr*, *rfbE* and SG can be observed, and their corresponding melting curves are depicted in Figure 1E. As can be observed, the dynamic range for both targets corresponds to six decimal dilutions, from 12.8 ng/μL to 0.13 pg/μL. The melting temperature was experimentally determined to be 77.7 ± 0.6°C and 73.0 ± 0.5°C for *ttr* and *rfbE*, respectively.

### Evaluation of the Methodology

#### Determination of the LOD_95_ for the DC-qPCR Combined with SpEn

The LOD_95_ was statistically calculated with the model described by Wilrich and Wilrich (2009). Similar values were obtained for both pathogens, 3.7 and 3.4 cfu/25 g for *Salmonella* spp. and *E. coli* O157, respectively, resulting in a combined LOD_95_ of 3.6 cfu/25 g for the method.

#### Fit-for-Purpose

The fit-for-purpose was determined in a total of 78 samples, 48 ground beef and 30 chicken breast. All parameters got values higher than 89% as only one ND was obtained for each pathogen. Both deviations were obtained in chicken samples. The results were confirmed by a culture-based approach, but it is worth to highlight that, while it was possible to isolate typical colonies of *Salmonella* spp. from XLD and ChromAgar *Salmonella*, in the case of *E. coli* O157, whenever high background microflora was present in the food samples, it was not possible to perform the confirmation with CT-SMAC, and only ChromAgar O157 allowed to isolate typical colonies. The specific values obtained, for each pathogen, as well as the combined results for the overall method, are summarized in Table 2.

**Table 2:** Summary of the fitness-for-purpose for each pathogen and the combined method.

Pathogen	N^*^	PD	NA	ND	PA	SE	SP	AC	PPV	NPV	*κ*	LOD_95_ (cfu/25 g)
*Salmonella* spp.	39	0	8	1	30	97	100	97	100	89	0.92	3.7
*Escherichia coli* O157	39	0	8	1	30	97	100	97	100	89	0.92	3.4
Combined results	78	0	16	2	60	97	100	97	100	89	0.92	3.6

*N*, total number of samples; PA, positive agreement; PD, positive deviation; NA, negative agreement; ND, negative deviation; SE, relative sensitivity; SP, relative specificity; AC, relative accuracy; PPV, positive predictive value; NPV, negative predictive value; *k*, Cohen’s kappa, interpretation: 0.81–1.00 almost complete concordance according to Altman (1991) and Anderson et al. (2011).

* The 78 samples corresponded to 48 ground beef and 30 chicken breast.

### Extended Study

#### Stressed Bacteria

In order to obtain some insights about the capacity of the novel methodology to detect the pathogens of interest in a real scenario, both bacteria were thermally stressed, and the spiked samples were stored under refrigeration for 24 and 48 h prior to analysis (four samples for each time point). The inoculation level for both pathogens was very low, 8 and 6 cfu/25 g of *E. coli* O157 and *Salmonella* spp., respectively. After 24 h of storage, three out of the four spiked samples were positive (75%). After 48 h, the number of positive samples for *Salmonella* spp. was still the same (three out of four, thus 75%), while only two were positive for *E. coli* O157 (50%).

#### Dead Bacteria

The novel method was also tested spiking some samples, eight in total, with different concentrations of dead bacteria (10^3^–10^6^ cfu/25 g) in order to determine the effect of inactivated target pathogens on the molecular method. Applying the statistical approach described by Wilrich and Wilrich (2009) for the LOD, it was calculated that 3.9 × 10^5^ and 2.9 × 10^5^ cfu/25 g of dead *Salmonella* spp. and *E. coli* O157 must be present in the original samples in order to obtain a PD linked to the presence of DNA from dead bacteria.

## Discussion

In the current study, a novel method combining short pre-enrichment (SpEn) in order to have a same-day detection, along with a novel detection approach named double chemistry qPCR (DC-qPCR) was developed and evaluated. The SpEn has been reported to provide good results for the pathogens included in the current study, *Salmonella* spp. and *E. coli* O157 (Fachmann et al., 2017; Garrido-Maestu et al., 2020), but no method was reported to attempt the simultaneous detection of both bacteria. In the current study, taking our previous findings dealing with the short-enrichment of *E. coli* O157 as a starting point, we have proceeded to tackle this issue by modifying the enrichment broth selected in order to select one more suited for both microorganisms. Regarding the DC-qPCR, we took advantage of the higher specificity provided by the hydrolysis probes, along with the capacity of SYBR Green I to bind double-stranded DNA and so to later perform melt curve analysis to confirm the results obtained. The combination of fluorescent probes with intercalating dyes has been previously described, but never tested for the detection of foodborne pathogens (Lind et al., 2006; Nagy et al., 2016).

Due to the fact that the final detection relies on DC-qPCR, first the effect of combining the different target primers and probes, along with those from a NC-IAC to rule out ND due to reaction inhibition, was assessed. The primers and probes selected to perform the multiplex detection of both pathogens, along with the NC-IAC, had been previously designed and tested by our research team. All three targets demonstrated to provide good results for their intended application in the corresponding original studies (detection of *Salmonella* spp., *E. coli* O157, and identify reaction inhibition) but were not tested in a multiplex format (Garrido-Maestu et al., 2018a,b, 2019, 2020). For this reason, we proceeded to re-evaluate them in this format. The multiplex amplification efficiency calculated for both targets was within range reported as acceptable (80–110%). Additionally, as expected, the implementation of the NC-IAC did not significantly affect, neither the amplification efficiency, nor the dynamic range covered for both targets. Finally, it was also confirmed that the peaks generated for the melt curve analysis were clearly distinguishable, thus suitable for the confirmation of each target (close to 5°C difference). Thus the DC-qPCR demonstrated to be a suitable alternative to simply detecting foodborne pathogens based on hydrolysis probes, as can provide the added value of melting curve confirmation of the results.

Due to the fact that the food industry in constantly seeking for novel and more rapid methods to detect different pathogens, the novel detection approach was combined with SpEn, which has already been described to allow for same-day detection of *Salmonella* spp. and *E. coli* O157 in different types of foodstuffs (Fachmann et al., 2017; Garrido-Maestu et al., 2020). The combined DC-qPCR with SpEn successfully allowed to accomplish the detection of both pathogens in one single working day, as after the initial pre-enrichment, the sample processing can be completed in roughly 1 h including the bacterial concentration, food left over proteolysis, sample clean-up, and DNA extraction. Additionally, economic cost of the sample treatment is relatively low as can be performed with a centrifuge, industrial proteases and the DNA extraction is performed by simple thermal lysis approach (Chelex). Furthermore, the results obtained in the process of evaluation (LOD_95_, the five quality parameters evaluated as well as the Cohen’s *k*) compared favorably to those previously described performing simplex pathogen detection. It is worth to highlight that the values calculated for SE (97%) and *k* (0.92) fulfill the requirements stated in the NordVal regulation for the validation of alternative methods (NordVaL, 2017). Overall, only two NDs were detected, one for each pathogen and both corresponded to chicken samples. The deviation related with *E. coli* O157 may be related with the fact that the inoculation level was very close to that of the LOD_95_ (calculated to be 5 cfu/25 g), while in the case of *Salmonella* spp., it was noticed that the sample causing the deviation got amplification by the specific probe, but classified as negative due to low Tm (below 77°C). This may have been originated by leftover contaminants from the actual food sample (chicken) or from the chemicals/reagents used in the process of sample pre-treatment and DNA extraction (e.g., the surfactant).

The last part of the evaluation study for the DC-qPCR combined with the SpEn is consisted in the determination of the capacity of the method to recover and detected stressed microorganisms, as well as to evaluate the capacity of dead bacteria to generate PD. In the particular case of stressed pathogens, it was observed that the number of deviations for *E. coli* O157 increases along with storage time, while for *Salmonella* spp. the number of positive and negative samples remained the same. Even though a relative viability of these pathogens has been reported when stored refrigerated, or even after stressing conditions in different food products (Park et al., 1970; Anassib et al., 2003; Barrera et al., 2007), it is worth to highlight that in the current study, the bacteria were submitted to two different stressing conditions, first heat and later cold storage, this treatment combined with the low initial concentration (<10 cfu/25 g) may be behind the reduced detection of stressed bacteria.

Finally, the effect of dead bacteria on the DC-qPCR was also determined as PD due to the presence of dead microorganisms has been commonly regarded as a major limitation for the implementation of these types of methodologies in the food industry (Postollec et al., 2011; Cangelosi and Meschke, 2014). It was statistically calculated that more than 10^5^ cfu of dead bacteria must be present in the original sample, in order to generate a PD with a 95% confidence, and more than 10^4^ for a 50% chance. It can be observed by these figures, food samples should be highly contaminated with dead bacteria in order to generate PD due to them, even though additional studies should be conducted, there are strong evidences to assume that only live pathogens will be detected following the described methodology. To the best of our knowledge, no other study has estimated the effect of dead bacteria in SpEn, but previous studies have investigated the capacity of other sample pre-treatments to remove free DNA. According to the studies of Mann et al. (2014) and Mayrl et al. (2009), application of the matrix lysis procedure allows to reduce 5 log the amount of free DNA, thus greatly reducing the possible interferences in the detection methodology. In the same way, the studies published by D’Urso et al. (2009) and Garrido-Maestu et al. (2018b) described a pre-treatment combined with filtration to remove dead microorganisms, and reported to be fully effective up to 10^4^–10^5^ cfu/25 g. A study from Wang et al. (2013), indicated that 10^6^ cells, or higher should be present in ground beef to generate positive result by qPCR due to dead microorganism. As it can be observed by the figures provided, the described method allowed to effectively detect viable bacteria in the same range of other previously published studies.

## Conclusion

In the present study, a novel qPCR approach combining the specificity of the hydrolysis probes, along with the capacity of the intercalating dyes for result confirmation, was developed. This technique was successfully combined with a SpEn approach for the multiplex detection of *Salmonella* spp. and *E. coli* O157. The final method was successfully evaluated on inoculated chicken and ground beef samples, and demonstrated capable of detecting thermally stressed bacteria, as well as avoiding false positive results due to the presence of dead bacteria.

## Data Availability Statement

The raw data supporting the conclusions of this article will be made available by the authors, without undue reservation.

## Author Contributions

AG-M envisioned the study, supervised the work, analyzed the data, and wrote the original draft of the manuscript. SA, JC, and FR conducted the experimental part. MP helped in the data analysis and revised the manuscript. All authors contributed to the article and approved the submitted version.

### Conflict of Interest

The authors declare that the research was conducted in the absence of any commercial or financial relationships that could be construed as a potential conflict of interest.
